# Temporal trends in semen quality at a single reproductive center in Shanghai, China, during 2021–2025 amid the COVID-19 pandemic

**DOI:** 10.3389/fendo.2026.1980263

**Published:** 2026-10-16

**Authors:** Shiyi Zhu, Jin Dong, Zhengmu Wu, Zhengquan Wang, Jun Yang, Chao Feng, Chunhua Tang, Yuchuan Zhou

**Affiliations:** 1 International Peace Maternity and Child Health Hospital, School of Medicine, Shanghai Jiao Tong University, Shanghai, China; 2 Shanghai Key Laboratory of Embryo Original Diseases, Shanghai, China

**Keywords:** COVID-19 pandemic, kinematic parameters, multiple linear regression model, semen quality, temporal trend

## Abstract

**Background:**

Accumulating epidemiological and andrological evidence has raised concerns about potential long-term declines in semen quality worldwide, highlighting the need for sustained, population-specific surveillance. In this study, we retrospectively analyzed temporal changes in semen quality during the period of 2021–2025 amid the COVID-19 pandemic. This work extends our previous report on annual trends in semen quality at the same center from 2015 to 2021.

**Methods:**

This retrospective study evaluated temporal changes in semen quality parameters among 21,932 men who attended a reproductive center in Shanghai between 2021 and 2025, a period characterized by widespread COVID-19 pandemic. After 2–7 days of sexual abstinence, semen samples were analyzed using computer-assisted sperm analysis to assess conventional semen parameters, including semen volume, sperm count, sperm concentration, and sperm motility, as well as sperm kinematic parameters, including curvilinear velocity, straight-line velocity, average path velocity, and beat-cross frequency. To better characterize temporal changes across different baseline semen-quality strata, participants were classified according to the WHO 2010 reference criteria into above-reference-limit and below-reference-limit groups. Differences among groups were assessed using one-way analysis of variance, the Kruskal–Wallis test, independent-samples t-test, and analysis of covariance, as appropriate. In the above-reference-limit group, multiple linear regression models including year, age, and abstinence duration were used to adjust for potential confounders and evaluate temporal trends in semen quality.

**Results:**

In the overall cohort and in both the above-reference-limit and below-reference-limit subgroups, sperm count, sperm concentration, and several CASA-derived kinematic parameters, including curvilinear velocity, straight-line velocity, average path velocity, and beat-cross frequency, generally showed increasing temporal trends from 2021 to 2025. After adjustment for age and abstinence duration, multiple linear regression analyses in the above-reference-limit group supported these associations. Semen volume, sperm count, sperm concentration, curvilinear velocity, straight-line velocity, average path velocity, and beat-cross frequency increased significantly over the study period, with regression coefficients of 0.0876, 4.815, 0.6929, 0.9423, 0.3553, 0.5150, and 0.1172, respectively. In contrast, sperm motility decreased significantly over time, with a regression coefficient of −0.7112.

**Conclusion:**

The observed increases in semen volume, sperm count, and sperm concentration contrast with the declining trends previously reported at the same reproductive center before the COVID-19 pandemic. These findings suggest widespread COVID-19 pandemic have reversed the annual changes in parameters related to semen quality.

## Introduction

Infertility is a major global health concern. Approximately one in six individuals of reproductive age experiences infertility during their lifetime, and male factors contribute to nearly half of these cases (1–3). Declining sperm quality has long been recognized as a major contributor to male infertility. In 1992, it was first reported that semen volume and sperm concentration had decreased by 50% over the period from 1930 to1991 (4). Since then, a growing body of evidence has described a gradual decline in semen quality over time (5–11). Moreover, the decline appears to have become more rapid and pronounced in the 21st century than in the preceding century (7). However, several studies have reported no significant temporal change or even an increase in selected parameters (12–15). In addition to conventional semen parameters, including semen volume, sperm count, sperm concentration, and sperm motility, computer-assisted sperm analysis (CASA) enables the simultaneous assessment of sperm kinematic parameters, such as curvilinear velocity (VCL), straight-line velocity (VSL), average path velocity (VAP), and beat-cross frequency (BCF). Our previous studies showed that conventional semen parameters declined from 2015 to 2021, whereas these kinematic parameters increased during the same period (8). Several other studies have also examined the clinical relevance and temporal trends of these parameters (16–18). Therefore, temporal changes in semen quality, encompassing both conventional and kinematic parameters, remain an important area of investigation.

Inconsistencies among studies assessing temporal changes in sperm quality raise important questions regarding their underlying determinants. Indeed, semen quality is widely recognized as a multifactorial phenotype influenced by multiple potentially modifiable factors, including environmental exposures (e.g., endocrine-disrupting chemicals, toxicants, and nano- and microplastics), medication use, lifestyle-related factors (e.g. obesity, nicotine use, excessive alcohol consumption, psychological distress), prolonged exposure to electromagnetic radiation, and infections (19–22). Among these influencing factors, the impact of viral infections including influenza, HIV and SARS-CoV-2 infections on semen quality is increasingly receiving attention (3, 23–25). Especially, since its emergence in December 2019, the COVID-19 pandemic has added a new dimension to the study of factors affecting sperm quality. Many studies have reported associations between SARS-CoV-2 infection and declines in multiple semen parameters, including semen volume, total motility, progressive motility, and the proportion of sperm with normal morphology (26–32). However, the impact of COVID-19 on sperm quality remains controversial. Other studies have reported no significant adverse effect on sperm quality, and some have observed higher values for selected semen parameters after infection (33–36). Several reports have further suggested that COVID-19 may have prolonged effects on semen quality lasting more than four months, particularly on sperm concentration and total motility (37–39). The COVID-19 pandemic created an urgent need for vaccines against SARS-CoV-2, and the potential reproductive effects of these vaccines have attracted substantial clinical and public health attention. Concerns have therefore been raised regarding their possible effects on male fertility. Recent studies have generally shown that COVID-19 vaccination does not adversely affect sperm quality or conventional semen parameters (40–46). Nevertheless, some studies have reported increases in semen volume, sperm concentration, and total sperm count after vaccination (47–49). Taken together, these heterogeneous findings highlight the complexity of evaluating the impact of SARS-CoV-2 infection and vaccination on male reproductive function.

Given the long-term decline in human fertility indicators and the impact of the COVID-19 pandemic, the present study examined changes in an integrative semen quality profile in potential relation to the COVID-19 pandemic in a large population by analyzing data from a single reproductive center over a past five-year period from 2021 to 2025 amid the COVID-19 pandemic. This integrated profile may provide a useful dataset for refining clinical reference standards for sperm kinematic parameters and offer additional insights into idiopathic male infertility.

## Materials and methods

### Study population and semen samples

This retrospective study included semen analysis records from the Reproductive Center of the International Peace Maternity and Child Health Hospital, Shanghai, China, collected between January 2021 and July 2025. A total of 29,246 semen samples were initially retrieved from the institutional database. The predefined eligibility criteria including male age between 18 and 60 years and reported abstinence duration between 2 and 7 days were applied to clean data. Only the first sample report was used when more than one sample from the same individual was available. Finally, 21,932 samples were retained for further analysis. The study population primarily represented men attending fertility-related clinical services, including infertility evaluation, assessment of oligoasthenospermia, and sperm freezing for assisted reproductive technology. Thus, this clinical context suggests a potential selection bias, as the cohort likely over-represents men with subfertility compared with a random population sample.

Only de-identified data extracted from existing medical records were used. No experimental intervention, treatment allocation, or additional biological sampling was performed. The requirement for informed consent was waived because of the retrospective and anonymized nature of the study. The investigation protocol was reviewed and approved by the Ethics Committee for Human Subjects of the International Peace Maternity and Child Health Hospital (GKLW-A-2026-064-01).

### Semen analysis

Semen samples were collected by masturbation and allowed to liquefy at 37 °C for at least 30 min before analysis. Semen volume was measured directly using a graduated collection vessel. Sperm concentration, sperm count, sperm motility, progressive motility, and kinematic parameters were assessed using a computer-assisted sperm analysis (CASA) system (Hamilton-Thorne, Beverly, MA, USA). The instrument settings were as follows: temperature: 35 °C; chamber depth: 20 μm; objective lens: 4; minimum contrast: 45; Maximum head area: 50 μm^2^; Minimum head area: 10 μm^2^; Minimum head brightness: 190; Minimum head brightness: 58; low VAP cutoff, 5 μm/s; low VSL cutoff, 5 μm/s; progressive VAP: 25 μm/s; progressive STR: 80. Thirty frames were acquired at a frame rate of 60 Hz. The playback feature was used during analysis to check the accuracy of the method. For each sample, a 5 µL aliquot was loaded into a standardized counting chamber (Leja Products B.V., Nieuw-Vennep, The Netherlands) and examined immediately to minimize post-liquefaction variability. At least 200 spermatozoa were evaluated across ten microscopic fields per chamber. All diagnostic semen assessments were conducted by two experienced technicians, and the results were reviewed and confirmed by a laboratory director with expertise in andrology. The same equipment, protocols, and trained personnel were used throughout 2021-2025. The recorded parameters included total sperm count (million), sperm concentration (million/mL), total motility (%), progressive motility (%), average path velocity (VAP, μm/s), straight-line velocity (VSL, μm/s), curvilinear velocity (VCL, μm/s), and beat-cross frequency (BCF, Hz). Spermatozoa with a path velocity exceeding 5 μm/s were classified as motile, whereas those with a path velocity greater than 25 μm/s and linearity greater than 80% were further categorized as progressively motile.

For comparative analysis, samples were stratified according to the reference thresholds in the fifth edition of the World Health Organization laboratory manual. Samples meeting all reference thresholds were assigned to the above-reference-limit (ARL) group (n = 10,504), whereas samples that did not meet at least one reference thresholds were assigned to the below-reference-limit (BRL) group (n = 11,428). The reference thresholds were semen volume ≥1.5 ml, total sperm count ≥39 million, sperm concentration ≥15 million/ml, total motility ≥40%, and progressive motility ≥32%. To examine demographic and pre-analytical trends, samples were also categorized by participant age group (18–24, 25–29, 30–34, 35–39, 40–44, and 45–60 years) and abstinence duration (2, 3, 4, 5, 6, and 7 days).

### Data analysis

Statistical analyses and data visualization were performed using GraphPad Prism (version 10.4.4; GraphPad Inc., San Diego, CA, USA) and R (version 4.2.2; R Foundation for Statistical Computing). Because conventional semen parameters and kinematic variables exhibited skewed and non-normal distributions, descriptive statistics are presented as medians with interquartile ranges. Comparisons across multiple groups were performed using the Kruskal–Wallis test, followed by the Mann–Whitney U test for pairwise comparisons when appropriate. To account for potential confounding by key determinants of semen quality, associations were further evaluated using analysis of covariance and multivariable regression models. Line graphs were generated using means with 95% confidence intervals (CIs) to visualize unadjusted parameter trends. Within the ARL group, multiple linear regression models were fitted to assess parameter changes while adjusting for calendar year, age, and abstinence duration. Model-based results were visualized using the ggplot2 package in R. Statistical significance was indicated by the following thresholds: *p < 0.05, **p < 0.01, ***p < 0.001, and ****p < 0.0001; p ≥ 0.05 was considered not statistically significant.

## Results

### Distribution of semen samples

A total of 21,932 semen samples were included in the present analysis. For comparative purposes, the cohort was stratified according to WHO 2010 reference limits into the above-reference-limit group (ARL; n = 10,504, 47.89%) and the below-reference-limit group (BRL; n = 11,428, 52.11%). The annual distribution of samples is shown in **Figure 1A**. Participant age and abstinence duration differed significantly across calendar years in the overall cohort and in each subgroup (all p < 0.001; **Figures 1B, C**). Rather than following a linear trajectory, age showed a rise-and-fall pattern over the study period, and a comparable non-monotonic pattern was observed for abstinence duration. In the total cohort, the median age was 32 years (IQR: 30–35), and the median abstinence duration was 4 days (IQR: 3–6), as summarized in **Table 1**.

**Figure 1:**
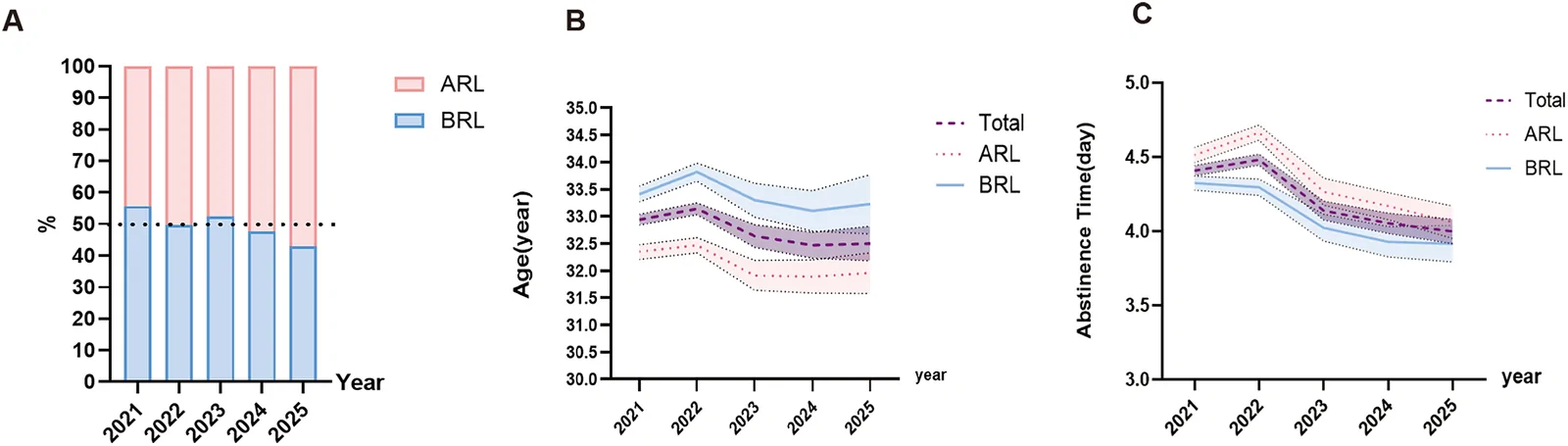
**S**ample distribution on type, age, and abstinence duration. **(A)** Proportions of the above reference limit group (ARL) and the below reference limit group (BRL). **(B, C)** Line charts of age **(B)**, and abstinence duration **(C)** based on means with 95% confidence intervals (CIs) over the years. The purple line represents the total sample, the pink line represents the ARL group, and the blue line represents the BRL group.

**Table 1:** Age and abstinence duration distribution in the total sample and the ARL and BRL groups over the years.

Year	n	Total sample		n	ARL group		n	BRL group	
		Age (year)	Abstinence duration (day)		Age (year)	Abstinence duration (day)		Age (year)	Abstinence duration (day)
2021	9569	32 (30,36)	4 (3,6)	4242	32 (29,35)	4 (3,6)	5327	32 (30,36)	4 (3,6)
2022	7811	32 (30,36)	4 (3,6)	3934	32 (29,35)	4 (3,6)	3877	33 (30,36)	4 (3,6)
2023	2068	32 (29,35)	4 (3,5)	985	32 (29,34)	4 (3,5)	1083	33 (30,36)	4 (3,5)
2024	1544	32 (29,35)	4 (3,5)	807	31 (29,34)	4 (3,5)	737	32 (29,36)	4 (3,5)
2025	940	32 (29,35)	4 (3,5)	536	31 (29,35)	4 (3,5)	404	32 (30,35.75)	4 (3,5)
P^α^		<0.0001	<0.0001		0.0001	<0.0001		<0.0001	<0.0001

Values are presented as median (interquartile range). ARL, above reference limit group; BRL, below reference limit group. p^α^, The Kruskal–Wallis test of the medians.

among the 5 years (2021–2025).

### Changes in conventional semen parameters

Annual medians and interquartile ranges for conventional semen parameters in the total cohort and the ARL group are presented in **Tables 2** and **3**, respectively. **Figures 2A–E** further display the corresponding means and 95% confidence intervals for the total cohort, ARL group and BRL group. In the unadjusted annual comparisons, sperm count (**Figure 2B**) and sperm concentration (**Figure 2C**) tended to increase during the later years of the observation period, whereas semen volume (**Figure 2A**), total motility (**Figure 2D**), and progressive motility (**Figure 2E**) showed no consistent annual changes in the descriptive analyses.

**Table 2:** Semen parameters in the total sample during 2021–2025.

Year	n	Semen volume (ml)	Sperm count (×10^6^)	Sperm concentration (×10^6^ ml^-1^)	Sperm motility (%)	Progressive motility (%)
2021	9569	2 (2-3)	77.24 (39.95-137.3)	34.92 (19.32-60.07)	47.2 (33.2-59.7)	38.35 (25.49-50.22)
2022	7811	2 (2-3.1)	70.43 (35.02-128.8)	29.99 (16.34-51.99)	52.7 (38.5-64.2)	44.95 (31.85-55.89)
2023	2068	2.6 (1.9-3.5)	75.74 (37.45-133.3)	29.1 (15.45-53.05)	47.25 (32.8-58.8)	40.84 (27.24-51.8)
2024	1544	2.3 (1.6-3.2)	90.39 (47.33-147.1)	39.31 (21.76-64.54)	48.5 (36.23-59.78)	41.5 (30.71-52.57)
2025	940	2.3 (1.7-3)	99.55 (59.78-160.1)	42.86 (25.3-69.18)	48.35 (37.53-59)	41.67 (31.98-51.3)
P^α^		<0.0001	<0.0001	<0.0001	<0.0001	<0.0001

Values are presented as median (interquartile range). p^α^, The Kruskal–Wallis test of the medians among the years.

**Table 3:** Semen parameters in the ARL group during 2021–2025.

Year	n	Semen volume (ml)	Sperm count (×10^6^)	Sperm concentration (×10^6^ ml^-1^)	Sperm motility (%)	Progressive motility (%)
2021	4242	2 (2-3)	110.3 (72.01-166.6)	44.69 (30.28-68.07)	57.5 (49.5-65.8)	48 (40.77-55.98)
2022	3934	2.5 (2-3.5)	106.8 (70.14-167.5)	40.35 (27.52-61.06)	60.45 (52.2-68.9)	51.88 (43.91-60.11)
2023	985	2.8 (2.1-3.6)	115.3 (78.8-168.7)	40.44 (27.37-61.9)	56.5 (48.8-64.4)	48.86 (41.65-56.65)
2024	807	2.5 (2-3.4)	129.6 (83.04-183.7)	47.25 (31.07-69.84)	55.6 (47.9-63.7)	48.41 (41.03-55.97)
2025	536	2.5 (2-3.3)	128.5 (84.56-190.2)	47.21 (34.11-72.65)	54.2 (47.33-62.08)	47.41 (40.48-54.79)
P^α^		<0.0001	<0.0001	<0.0001	<0.0001	<0.0001

Values are presented as median (interquartile range). p^α^, The Kruskal–Wallis test of the medians among the years.

**Figure 2:**
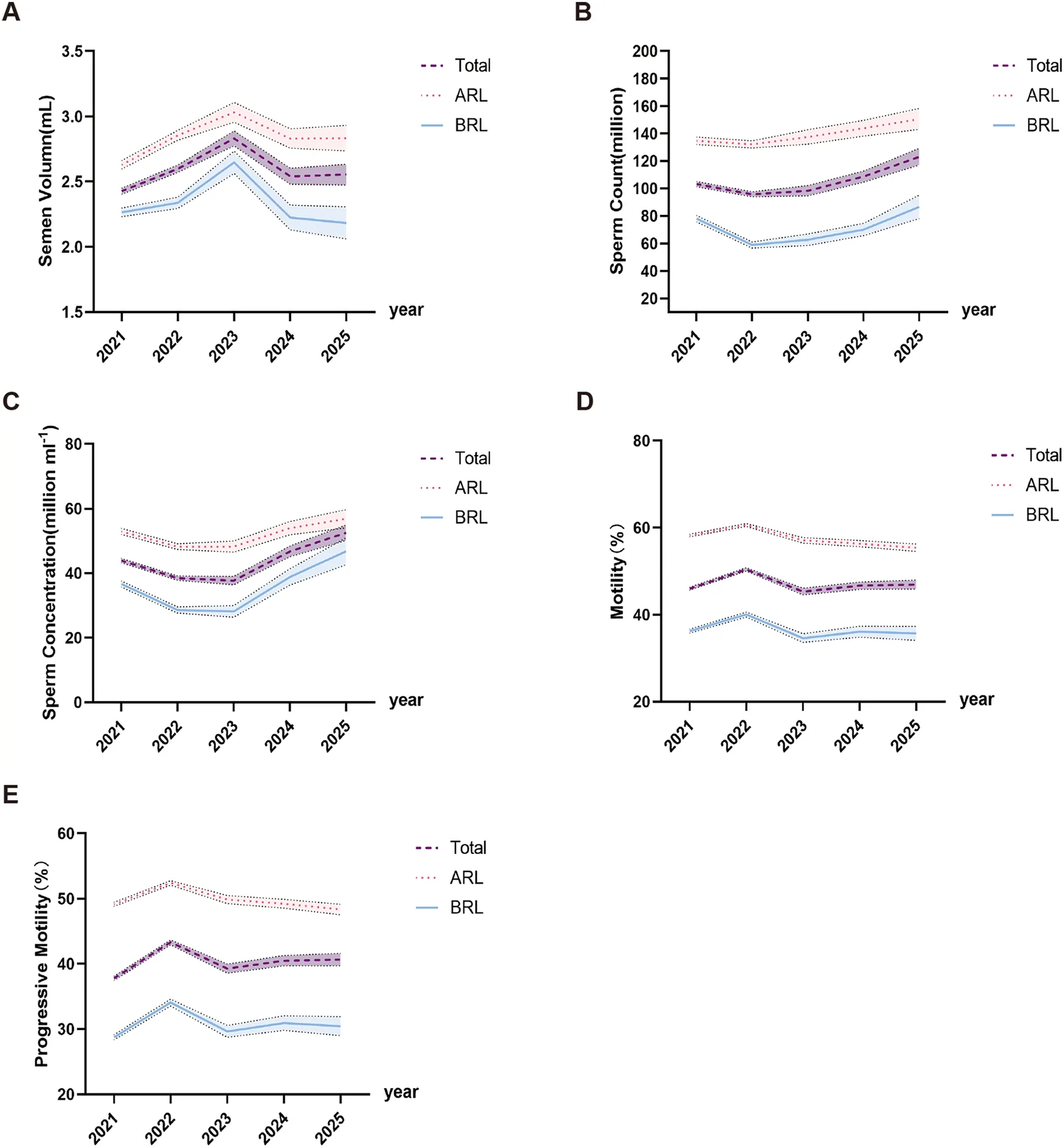
Line charts of semen parameters in the three type groups. Means and 95% CIs of semen volume **(A)**, sperm count **(B)**, sperm concentration **(C)**, motility **(D)**, and progressive motility **(E)** in the groups over the years. The purple line represents the total sample, the pink line represents the ARL group, and the blue line represents the BRL group.

### Changes in sperm kinematic parameters

Annual changes in sperm kinematic parameters are detailed in **Tables 4** and **5** for the total cohort and the ARL group, respectively. **Figures 3A–D** summarizes the means and 95% confidence intervals of VCL, VSL, VAP, and BCF across the total cohort, ARL group, and BRL group. Although these CASA-derived parameters fluctuated across individual years, VCL, VSL, VAP, and BCF showed overall upward trajectories from the beginning to the end of the study period.

**Table 4:** Sperm kinematic parameters of the total sample during 2021–2025.

Year	n	VCL (μm s^-1^)	VSL (μm s^-1^)	VAP (μm s^-1^)	BCF (Hz)
2021	9569	107.1 (90.59-124.4)	61.73 (52.72-69.82)	71.6 (61.63-80.84)	27.66 (25.34-29.92)
2022	7811	113.5 (97.34-130.3)	63.8 (55.6-71.55)	74.79 (65.51-83.41)	27.46 (25.14-29.74)
2023	2068	109.1 (94.14-124.6)	62.03 (54.15-69.28)	72.52 (63.95-80.85)	27.27 (24.89-29.75)
2024	1544	112.7 (98.26-126.9)	64.07 (55.84-70.98)	74.97 (66.44-82.23)	27.88 (25.61-30.05)
2025	940	113.5 (99.5-128.4)	65.08 (57.39-72.59)	75.7 (67.71-83.88)	28.51 (25.94-30.77)
P^α^		<0.0001	<0.0001	<0.0001	<0.0001

Values are presented as median (interquartile range). p^α^, The Kruskal–Wallis test of the medians among the years.

**Table 5:** Sperm kinematic parameters in the ARL group during 2021–2025.

Year	n	VCL (μm s^-1^)	VSL (μm s^-1^)	VAP (μm s^-1^)	BCF (Hz)
2021	4242	113.9 (100.4-129.1)	65.92 (59.11-72.86)	76.71 (68.97-84.34)	28.04 (26.02-29.98)
2022	3934	118.4 (104.5-133.8)	66.92 (60.41-73.35)	78.16 (70.86-85.49)	28.02 (26-30.03)
2023	985	115 (101.4-130.8)	65.49 (59.57-71.83)	76.52 (69.8-83.57)	28.08 (25.78-30.07)
2024	807	116.9 (104.9-131.1)	66.59 (60.87-72.75)	77.75 (72.19-84.35)	28.34 (26.16-30.24)
2025	536	120 (106.7-133.1)	68.61 (62.27-75.31)	79.55 (73.77-86.67)	28.83 (26.4-30.84)
P^α^		<0.0001	<0.0001	<0.0001	<0.0001

Values are presented as median (interquartile range). p^α^, The Kruskal–Wallis test of the medians among the years.

**Figure 3:**
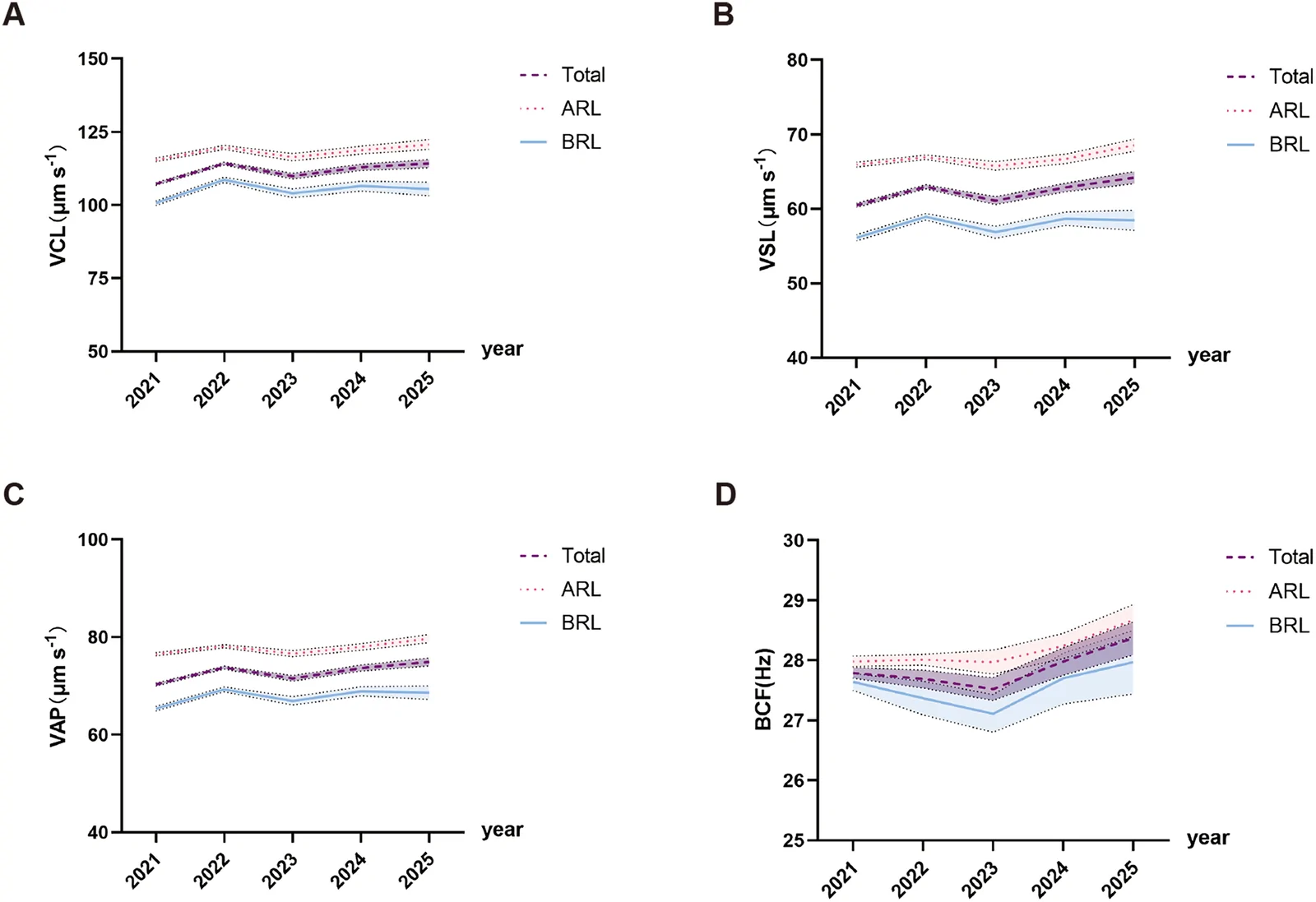
Line charts of kinematic parameters in the three type groups. Means and 95% CIs of VCL **(A)**, VSL **(B)**, VAP **(C)**, and BCF **(D)** in the groups over the years. The purple line represents the total sample, the pink line represents the ARL group, and the blue line represents the BRL group.

After adjustment for age and abstinence duration, all four kinematic parameters remained significantly higher in the ARL group than in the BRL group (all p < 0.0001; **Table 6**). This difference indicates that sperm kinematic indices provide additional quantitative information on motility performance and may complement conventional motility-based semen assessment.

**Table 6:** Variation analysis of kinematic parameters in the different groups.

Parameter	Total Sample n=21932	ARL n=10504	BRL n=11428	P^α^	P^β^
VCL (μm s^-1^)	110.2 (94.12-126.9)	116.4 (102.8-131.6)	103.3 (86.27-121.8)	<0.0001	<0.0001
VSL (μm s^-1^)	62.87 (54.25-70.64)	66.45 (59.94-73.06)	58.39 (48.94-66.95)	<0.0001	<0.0001
VAP (μm s^-1^)	73.37 (63.76-82.07)	77.52 (70.22-84.88)	68.28 (57.68-77.9)	<0.0001	<0.0001
BCF (Hz)	27.61 (25.26-29.89)	28.11 (26.03-30.07)	27.08 (24.51-29.67)	<0.0001	<0.0001

Values are presented as median (interquartile range). p^α^, The Mann-Whitney U test of the kinematic parameters between the ARL and BRL groups. p^β^, Covariance analysis of the kinematic parameters between the ARL and BRL groups, adjusted for age and abstinence duration.

### Multiple linear regression models in the ARL group

Consistent with our laboratory’s previous findings (8), age and abstinence duration were identified as independent factors associated with semen parameters. Therefore, subsequent regression analyses were adjusted for these two variables to reduce potential confounding. The distribution of age and abstinence duration across subgroups, together with their associations with semen parameters, are provided in the **Supplementary Materials** (**Supplementary Figures S1**–**S4**).

Multiple linear regression models were then used to evaluate the associations between calendar year, age, and abstinence duration, and semen parameters in the ARL group (**Table 7**). After adjustment, semen volume (β1 = 0.08759; CI: 0.06836, 0.1068; **Figure 4A**), sperm count (β1 = 4.815; CI: 3.385, 6.246; **Figure 4B**), and sperm concentration (β1 = 0.6929; CI: 0.1948, 1.191; **Figure 4C**) increased significantly over time. In contrast, total motility declined significantly over the study period (β1 = −0.7112; CI: −0.8945, −0.5279; **Figure 4D**), whereas progressive motility showed a nonsignificant downward tendency (β1 = −0.1056; CI: −0.2821, 0.07087; **Figure 4E**; p = 0.2408). The kinematic parameters displayed a different pattern, with significant annual increases in VCL (β1 = 0.9423; CI: 0.5790, 1.306; **Figure 4F**), VSL (β1 = 0.3553; CI: 0.1844, 0.5262; **Figure 4G**), VAP (β1 = 0.515; CI: 0.3304, 0.6995; **Figure 4H**), and BCF (β1 = 0.1172; CI: 0.06645, 0.1679; **Figure 4I**; all p < 0.0001). The estimates indicate that, within the ARL group, semen volume increased by 0.08759 mL per year, sperm count by 4.815 × 10^6^ per year, and sperm concentration by 0.6929 × 10^6^ mL per year. Total motility decreased by 0.711 percentage points per year, while progressive motility decreased by 0.1056 percentage points per year, although the latter association was not statistically significant. VCL, VSL, VAP, and BCF increased annually by 0.9423 µm/s, 0.3553 µm/s, 0.515 µm/s, and 0.1172 Hz, respectively.

**Table 7:** Multiple regression linear model of semen parameters.

	Predictors	Semen volume Estimates (95%CI)	Sperm count Estimates (95%CI)	Sperm concentration Estimates (95%CI)	Total motility Estimates (95%CI)	Progressive motility Estimates (95%CI)
Semen parameters	Year (β1)	0.08759**** (0.06836,0.1068)	4.815**** (3.385,6.246)	0.6929** (0.1948,1.191)	-0.7112**** (-0.8945,-0.5279)	-0.1056 (-0.2821,0.07087)
	Age (β2)	-0.02135**** (-0.02619,-0.01652)	-0.2209 (-0.5806,0.1388)	0.336**** (0.2107,0.4613)	-0.2023**** (-0.2484,-0.1562)	-0.2189**** (-0.2633,-0.1745)
	Abstinence duration (β3)	0.09865**** (0.08533,0.1120)	14.05**** (13.06,15.04)	3.551**** (3.206,3.896)	-0.2816**** (-0.4086,-0.1547)	-0.3982**** (-0.5204,-0.2759)
	R2	0.03147	0.07018	0.04066	0.01363	0.01313
	Predictors	VCL Estimates (95%CI)	VSL Estimates (95%CI)	VAP Estimates (95%CI)	BCF Estimates (95%CI)	
Semen parameters	Year (β1)	0.9423**** (0.5790,1.306)	0.3553**** (0.1844,0.5262)	0.515**** (0.3304,0.6995)	0.1172**** (0.06645,0.1679)	
	Age (β2)	-0.3067**** (-0.3981,-0.2153)	-0.091**** (-0.1340,-0.04802)	-0.1408**** (-0.1872,-0.09441)	-0.005583 (-0.01834,0.007177)	
	Abstinence duration (β3)	-1.047**** (-1.299,-0.7958)	-0.2606**** (-0.3790,-0.1423)	-0.4373**** (-0.5651,-0.3095)	0.03855* (0.003410,0.07369)	
	R2	0.0141	0.005527	0.01154	0.002336	

Multiple linear regression model of sperm analysis parameters. Values were presented as regression coefficient (Estimates) with 95% confidence interval (95% CI). Year, age, and abstinence duration were included as factors. β1, β2, and β3 represent the slope of liner regression by year, age, and abstinence duration, respectively, weighted by the other two factors. R^2^, R-squared represented the fitness of the model. Significance was indicated as p < 0.05 (*), p < 0.01 (**), p < 0.001 (***) and p<0.0001(****).

VCL (p < 0.0001), VSL (p < 0.0001) and VAP (p < 0.0001) declined with age.

**Figure 4:**
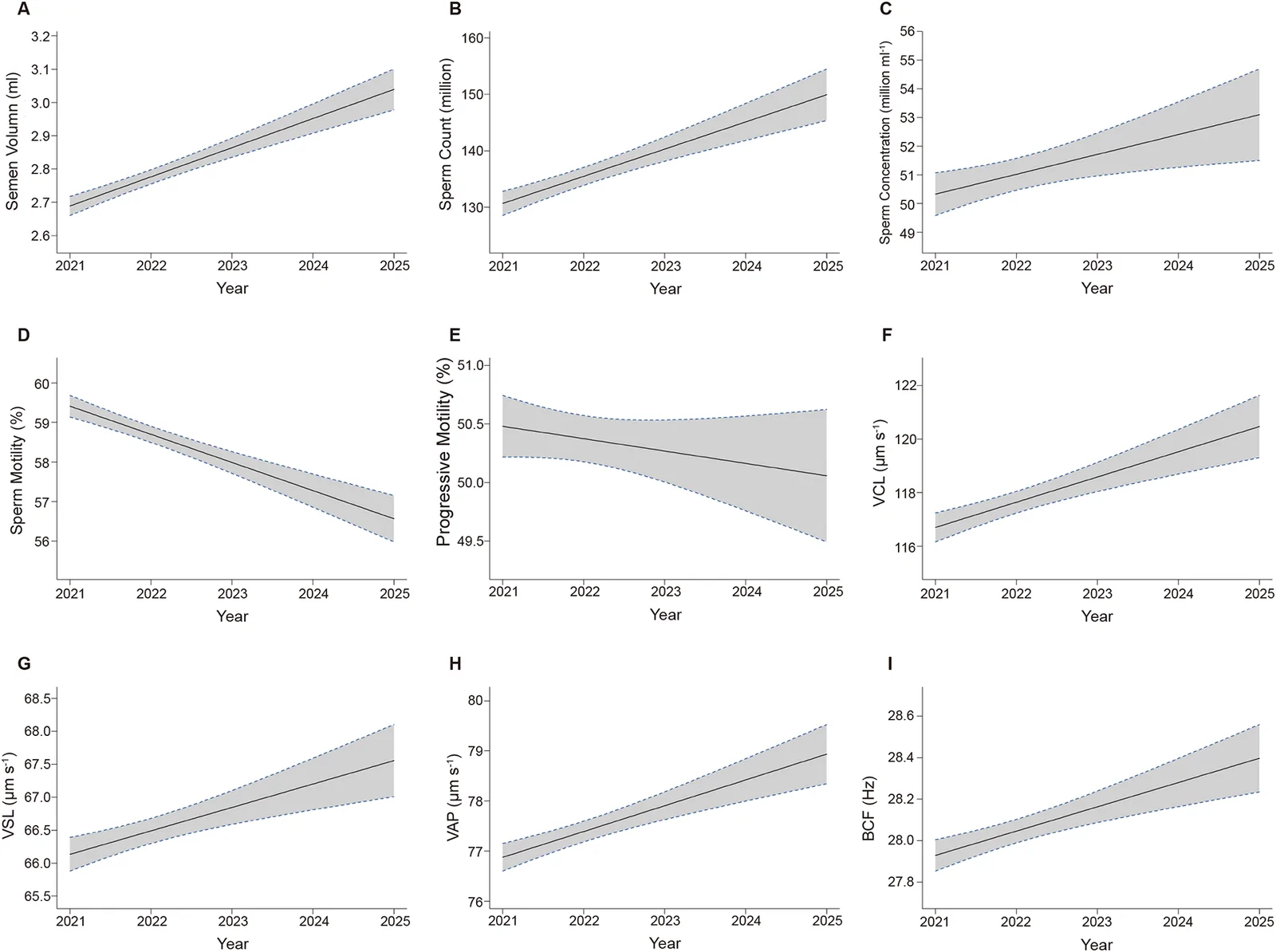
The plotting of multiple regression linear model of semen and kinematics parameters by year. Images of multiple regression linear models fitting of semen parameters: semen volume **(A)**, sperm count **(B)**, sperm concentration **(C)**, motility **(D)**, and progressive motility **(E)** and kinematics parameters: VCL **(F)**, VSL **(G)**, VAP **(H)**, and BCF **(I)** by year adjusted for age and abstinence duration.

## Discussion

Sperm quality trends over time remain an important and debated clinical issue. Most epidemiological surveys and observational studies have reported declines in semen quality over recent decades (1, 2, 4–11). However, other studies have found no significant temporal changes or even improvements in selected semen parameters (12–15). These inconsistencies may reflect differences in study populations, geographical regions, environmental exposures, and methodological approaches. Our earlier work demonstrated significant decline in conventional semen parameters, including semen volume, sperm count, sperm concentration and sperm motility during 2015–2021, and the present study extended the observation period to 2021–2025 at the same reproductive center. In this investigation, we analyzed 21,932 semen samples collected from the reproductive center between 2021 and 2025, a period encompassing widespread COVID-19 pandemic. We found that semen volume, sperm count and sperm concentration have been increasing annually, which is significantly opposite to the downward trend we observed at the same reproductive center from 2015 to 2021; whereas the decline in sperm motility and increase in kinematic parameters year by year are similar to our previous reports (8). The coexistence of increasing sperm quantity-related parameters and improving kinematic velocity indices with declining sperm motility suggests that different dimensions of semen quality may not change in parallel. This finding highlights the importance of integrating conventional semen parameters with CASA-derived kinematic measures when evaluating temporal changes in male reproductive health. The divergence in conventional parameters of semen quality between these two consecutive time periods may be due to the impact of the COVID-19 outbreak. That is to say, COVID-19 pandemic may have a long-term impact on the trajectory of some semen-quality parameters.

After adjustment for age and abstinence duration, our data confirmed the consensus finding of decreased sperm motility, which is consistent with both the broader literature and our previous observations. We also found that sperm kinematic parameters, including VCL, VSL, VAP, and BCF, increased from 2021 to 2025, consistent with our previous finding at the same center (8). Sperm velocity is closely related to motility state and is used in CASA to characterize the movement patterns of motile spermatozoa; it also increases during capacitation and hyperactivation. The inverse pattern of declining sperm motility but increasing kinematic parameters may be partly explained by the distinct definitions of these parameters. The motility parameter reflects the proportion of spermatozoa exhibiting motility, whereas VCL, VSL, VAP, and BCF quantify movement characteristics, including velocity patterns and flagellar beating, within the sperm subpopulation. Thus, slower or poorly motile spermatozoa may become classified as immotile or be underrepresented in kinematic calculations, resulting in higher average velocities among the remaining motile cells. Beyond this methodological explanation, another possibility is that the improved kinematic values among motile spermatozoa may represent a compensatory response that partially offsets the decline in proportion of motile cells. However, as suggested by our previous work, aberrant or premature sperm hyperactivation cannot be excluded. The mechanisms underlying these dissociated changes in sperm motility and kinematic parameters remain insufficiently understood. Future longitudinal studies incorporating advanced sperm functional assays are therefore needed.

There may be several potential reasons for the significant changes in semen quality parameters in the same reproductive center after the outbreak of COVID-19. This shift may include the direct or indirect effects of SARS-CoV-2 infection, COVID-19 vaccination and pandemic-related lifestyle changes in the study population. Firstly, the detection of SARS-CoV-2 in the testes and semen of infected individuals suggests the possibility of direct local effects on sperm cells (50–52). SARS-CoV-2 infection has also been associated with oxidative stress in the seminal environment, as reflected by increased levels of reactive oxygen species (ROS) (53). Such oxidative stress may be particularly detrimental to human spermatozoa (54). Several studies have reported that SARS-CoV-2 infection is associated with declines in multiple semen parameters, including semen volume, total motility, progressive motility, and the proportion of sperm with normal morphology (26–32). The effects of COVID-19 vaccination on sperm quality have also been reported. Recent studies have reported increases in total sperm count, sperm concentration, and semen volume after vaccination (47–49). In addition, sperm motility-related kinematic parameters, including VCL, VAP, VSL, and BCF, have been reported to differ before and after vaccination (55). In the present study, the fluctuation in semen quality from 2021 to 2023 and the subsequent trends through 2025 differed from those observed during 2015-2021. These findings suggest that COVID-19 pandemic may have contributed to changes in the long-term trajectory of semen quality. Secondly, lifestyle changes during lockdowns may also have influenced semen quality. The COVID-19 prevention policy was accompanied by substantial changes in behavior and societal activity, including psychological stress, altered daily routines, increased sedentary behavior, widespread mask use, and reduced outdoor activity due to social distancing mandates and lockdown measures (56). The effects of these changes on semen quality are likely to be bidirectional and complex. On the one hand, reduced physical activity and altered dietary patterns, such as irregular meal timing and increased portion sizes, may increase the risk of weight gain and metabolic deterioration (57, 58). These conditions may impair spermatogenesis, consequently leading to declines in sperm concentration, motility, and overall health (59–61). On the other hand, lockdown periods may have been associated with some favorable behavioral changes. Survey-based studies have reported improvements in dietary habits after the COVID-19 outbreak, including increased consumption of home-prepared and nutrient-rich foods (62). This shift is clinically relevant, as higher intake of fruits and vegetables has been associated with improved semen parameters (63). Therefore, the dissociated pattern observed in our study, characterized by an increase in semen volume, sperm count, and sperm concentration alongside a decrease in sperm motility, may reflect a complex interplay among viral exposure, lifestyle-mediated effects, and possible compensatory physiological responses.

This study has several strengths. First, from a methodological perspective, we used a multiple linear regression model adjusted for age and abstinence duration, which improved the robustness of the estimated temporal trends. Second, from an analytical perspective, the inclusion of multiple sperm kinematic parameters, such as VCL and VSL provided a more detailed functional assessment than conventional semen parameters alone. Third, we identified concurrent increases in conventional quantitative parameters, including semen volume, sperm count, and sperm concentration, and in kinematic velocities from 2021 to 2025; this pattern differs from some previous reports of stable or declining trends and highlights the complexity of temporal changes in semen quality. Fourth, the observation period covered the peak and post-peak phases of the COVID-19 pandemic, providing a relevant dataset for exploring potential longer-term associations between the COVID-19 pandemic period and sperm function.

This study has also several limitations that should be considered when interpreting the findings. First, the generalizability of the results is limited because the data were obtained from a single reproductive center and included both apparently healthy men and men seeking fertility evaluation; therefore, the observed trends may not fully represent semen-parameter trends in the general population. In addition, the number of observations decreases substantially over the study period. This is mainly due to the opening of a new branch hospital and the renovation of the old hospital area, which led to the referral of participants who came to our reproductive center. Second, several important factors may influence semen quality, including fever, COVID-19 severity, lifestyle, smoking, obesity, environmental exposures, medications, and changes in the patient population. At present, our investigation lacks individual-level COVID-19 and vaccination information of donors, which limits our further interpretation. Thus, the relationship between the change trend of semen quality and the COVID-19 pandemic is only indirectly inferred Third, although age and abstinence duration were adjusted for, residual confounding by unmeasured factors, such as lifestyle factors and environmental exposures, cannot be excluded. Finally, the clinical significance of the observed changes in kinematic parameters remains uncertain.

## Conclusion

Our present results from a single center in Shanghai clearly indicate that semen volume, sperm count, sperm concentration, and sperm kinematic parameters have shown an increasing trend from 2021 to 2025, while sperm motility and progressive motility continued to decrease annually. The increasing trends in semen volume, sperm count and sperm concentration are opposite to the decreasing trends observed at the same center before and during the COVID-19 outbreak. To our knowledge, this is the first study to investigate the potential long-term association between the COVID-19 pandemic and semen quality.

## Data Availability

The original contributions presented in the study are included in the article/**Supplementary Material**. Further inquiries can be directed to the corresponding authors.
